# Replacement of Antarctic krill (*Euphausia superba*) by extruded feeds with different proximate compositions: effects on growth, nutritional condition and digestive capacity of juvenile European lobsters (*Homarus gammarus*, L.)

**DOI:** 10.1017/jns.2021.27

**Published:** 2021-05-12

**Authors:** Renata Goncalves, Manuel Gesto, Maria Alexandra Teodósio, Vânia Baptista, Carmen Navarro-Guillén, Ivar Lund

**Affiliations:** 1 Technical University of Denmark, DTU Aqua, Section for Aquaculture, The North Sea Research Centre, 9850 Hirtshals, Denmark; 2 CCMAR – Centre of Marine Sciences, University of Algarve, Campus de Gambelas, 8005-139 Faro, Portugal

**Keywords:** Digestive enzymatic activity, Feed efficiency, Macronutrients, Nucleic acid indices

## Abstract

Extruded feeds are widely used for major aquatic animal production, particularly for finfish. However, the transition from fresh/frozen to extruded/pelleted feeds remains a major obstacle to progressing sustainable farming of European lobster (*Homarus gammarus*). The aim of the present study was to investigate the effects of using extruded feeds with different protein levels and lipid/carbohydrate ratios on growth, feed utilisation, nucleic acid derived indices (sRD) and digestive enzymatic activity of *H. gammarus* juveniles. Six extruded feeds were formulated to contain two protein levels (400 and 500 g/kg), with three lipid/carbohydrate ratios (LOW – 1:3; MEDium – 1:2; HIGH – 1:1). The extruded feeds were tested against Antarctic krill (*Euphausia superba*) used as control (CTRL). Overall, the CTRL and 500MED feed supported the highest growth and nutritional condition estimated by means of sRD, while the poorest results were observed for the 400HIGH and 400MED groups. The FCR was significantly lower in the CTRL than all extruded feeds, among which the most efficient, i.e., lower FCR, was the 500MED. The highest activity of trypsin and amylase in lobsters fed the 400MED and 400HIGH feeds points to the activation of a mechanism to maximise nutrients assimilation. The highest lipase activity observed for the 500LOW and 500MED groups indicates a higher capacity to metabolise and store lipids. Overall, the results suggest that the 500MED feed (500 g/kg protein, 237 g/kg carbohydrates and 119 g/kg lipids) is a suitable extruded feed candidate to replace Antarctic krill, commonly used to grow lobster juveniles.

## Introduction

The European lobster (*Homarus gammarus*, L.) is an economically important decapod crustacean geographically ranging from Northern Norway to Morocco and the Eastern Mediterranean^(1)^. The increased fishing pressure from 1930 to 1970 led to the collapse of several European lobster populations throughout Europe^(2)^. To counteract the decline, multiple experimental stock enhancement programmes have been launched to release the large numbers of juveniles to lobster grounds in France^(3)^, the United Kingdom^(4)^, Ireland^(5)^, Norway^(6)^ and Germany^(7)^. Commercial aquaculture is gaining interest as an additional approach. However, in comparison with other aquaculture sectors, lobster farming still faces several constraints for their commercial production to be economically sustainable. Among them are the high mortality rates, energy and feeding costs^(8)^. Significant advances in automated rearing systems and water quality have been made in recent years that improve European lobster hatchery production and cost-effectiveness^(9–13)^. Nevertheless, the lack of a balanced artificial feed that is affordable, with better nutritional value, and easier to handle and store compared to live, fresh or frozen foods remains a major drawback in the sustainable farming of *H. gammarus*^(14,15)^.

Fresh, live mussels^(16,17)^ and frozen krill^(18,19)^ are commonly used as preferred reference diets, as they appear to perform consistently well as food for several lobster species. However, the future success of lobster aquaculture will depend on the ability to replace live, fresh or frozen diets by more practical and cost-effective artificial feeds. The transition to specifically formulated, dry pelleted feeds has been applied with relative good success to several crustacean species within the Penaeidae^(20)^. While considerable research efforts in the last three decades have been devoted to spiny lobster species (Palinuridae), the transition to extruded feeds has proven to be challenging^(21,22)^. Results indicate that protein is the most important macronutrient for optimal growth. Carbohydrates are readily digested and absorbed but their subsequent fate is poorly understood, while lipids appear to be poorly digested, absorbed and used. These findings are in agreement with the chemical composition of the preferred prey of several spiny lobster species which presents high protein, low lipid and moderate to high carbohydrate content^(23)^.

The feasibility of using artificial feeds for the production of juvenile American lobster (*Homarus americanus*) was extensively investigated^(16,24–27)^, but growth obtained was not as high as animals that are fed live/fresh food. The efforts on the development of artificial feeds for the European lobster have been less and only a few published studies refer to the potential application of formulated feeds for juvenile *H. gammarus* farming^(18,28)^. Reported results are similar to what has been observed for the *H. americanus*, i.e., lower growth performance in lobsters fed the experimental artificial diets. The relatively limited success of formulated feeds for lobster juveniles can be partially explained by the lack of information on how lobsters digest and assimilate formulated feeds^(22)^. Additionally, the higher growth and feeding rates of lobsters fed natural diets has been generally related to: (a) their superior water stability and attractiveness and (b) a nutrient profile approximating closely to their body composition^(29)^.

Most nutritional studies performed with crustacean species address weight and length gains as growth performance indicators. However, maximum size *per se* does not necessarily imply the best condition. This is particularly relevant in decapod crustacean species since their body weight is highly influenced by the water content and moulting stage^(30)^. The use of biochemical indicators can aid in a more comprehensive evaluation of the nutritional status of the reared animals. The ratio of RNA:DNA concentrations has been previously used as an indicator of recent growth and nutritional condition in a variety of crustacean species including the *H. gammarus*^(31–34)^. The main premises are that the amount of RNA in a cell varies in proportion to protein synthesis, while the amount of DNA remains fairly constant^(35)^. Thus, theoretically, poor nutritional condition contributes to low protein synthesis and slow growth, resulting in a low RNA:DNA ratio^(36)^.

Increasing the knowledge on the activity of digestive enzymes of European lobsters is essential to establish a better understanding of their digestive ability. Analysing the digestive response might help revealing why some diets show better performance than others might. There are few published studies referring to the digestive capacity of *H. gammarus*. A range of carbohydrases^(37)^ and proteases^(38)^ have been detected in the hepatopancreas and gastric juice of wild-caught adults. However, changes in the digestive enzyme activity with the type of food or nutritional composition have not yet been determined in European lobster. To the best of our knowledge, only one such study on lobsters has been published^(39)^ but on a different species. In this cited work, the rearing of juvenile spiny lobsters (*Jasus edwardsii*) on a formulated diet for 6 months resulted in a pronounced decrease in the enzymatic activity (protease, trypsin, α-amylase and α-glucosidase) of the foregut and digestive gland compared with juveniles reared on fresh mussels.

In the present study, we aimed to evaluate the effect of protein levels and the ratios between lipid and carbohydrates in formulated extruded feeds in terms of survival, growth, feed utilisation, nutritional condition and enzymatic activity to explore the potential nutritional requirements of European lobster juveniles. The experimental extruded feeds were tested against a conventional control diet (Antarctic krill, *Euphausia superba*).

## Material and methods

### Experimental animals

Juveniles of *H. gammarus* were obtained from a wild European lobster female caught in the Skagerrak coast of North Jutland, Denmark. Animals used in this experiment were procured from the same female to reduce phenotypic variation. The fertility/fecundity of the chosen female, as well as the survival and apparent quality of her progeny, was observed to be as usual in our facilities. From hatching and until settling, larvae were reared in 500-l rectangular tanks installed at the North Sea Oceanarium, Hirtshals. Each tank was equipped with a top seawater inflow (50 l/h) and a vertical outflow filter (1 mm mesh diameter). Temperature and salinity were kept at 18 ± 1°C and 34 ± 1 PSU, respectively. Strong aeration was provided from the bottom of the tank using airstones to maintain larvae in the water column. Larvae were stocked sequentially into tanks over three consecutive days after hatching at an initial density of 5–7 larvae/l and daily fed thawed Antarctic krill, *Euphausia superba* (Akudim A/S, Denmark). The bottom of the tanks was syphoned every week to remove debris. Upon metamorphosis to stage IV^(40)^, post-larvae were transferred to the aquaculture facilities at the Technical University of Denmark, Section for Aquaculture, Hirtshals. Animals were individually reared in 3D printed polylactic acid (PLA) bioplastic cassette systems of 200 ml compartments with perforated grids for water in- and outlet. Cassettes were distributed and immersed in raceway tanks of 80-l capacity supplied by the recirculation seawater system at a constant flow rate of 330 l/h (18 ± 0⋅5°C temperature, 34 ± 1 PSU salinity, >90 % dissolved oxygen, <0⋅1 mg/l ammonia-N) subjected to a photoperiod cycle of 8 h light : 16 h dark. Lobsters were adapted to the trial conditions for 1 month, during which they were daily fed thawed Antarctic krill.

### Experimental extruded feeds and control diet

Details of the experimental feeds and control diet are reported in Ref. (18). Briefly, experimental extruded feeds were formulated to contain 400 or 500 g/kg protein, with lipid contents ranging from 86 to 233 g/kg and carbohydrates from 210 to 347 g/kg resulting in three ratios of lipid to carbohydrate: low (1:3), medium (1:2) and high (approx. 1:1). This resulted in six experimental feeds referred to as 400LOW, 400MED, 400HIGH, 500LOW, 500MED and 500HIGH. The different protein, carbohydrate and lipid contents were achieved by altering squid meal, wheat gluten, wheat starch and fish oil inclusion levels. Experimental feeds were extruded as 4 mm pellets and were manufactured by SPAROS, Lda. (Olhão, Portugal). Thawed Antarctic krill was used as control feed (CTRL). Proximate analysis of the Antarctic krill and extruded feeds were performed in duplicate. Feeds and krill were finely ground using a Krups Speedy Pro homogenizer and analysed for crude protein (i.e. Kjeldahl N × 6⋅25^(41)^), total lipid^(42)^, dry matter (DM) and ash^(43)^. Likewise, amino acid analyses were performed in duplicate by use of hydrolysed feed samples^(44)^. The amino acid content was determined by HPLC^(45)^. The ingredients and proximate chemical composition of the experimental extruded feeds and control diet are shown in Table 1. The amino acid profiles of the extruded feeds and control diet are listed in Table 2.

**Table 1.** Formulation and chemical composition of experimental extruded feeds and Antarctic krill (adapted from^(18)^)

Protein level	400 g/kg		500 g/kg				
L:CHO ratio	LOW	MED	HIGH	LOW	MED	HIGH	CTRL
*Ingredients (g*/*kg)*							
Antarctic krill							1000⋅0
Fish meal^a^	150⋅0	150⋅0	150⋅0	150⋅0	150⋅0	150⋅0	
Squid meal^b^	125⋅0	125⋅0	125⋅0	255⋅0	255⋅0	255⋅0	
Krill meal^c^	250⋅0	250⋅0	250⋅0	200⋅0	200⋅0	200⋅0	
Wheat gluten^d^	20⋅0	20⋅0	20⋅0	50⋅0	50⋅0	50⋅0	
Wheat meal^e^	172⋅5	172⋅5	172⋅5	172⋅5	172⋅5	171⋅5	
Wheat starch^f^	229⋅0	171⋅0	89⋅0	141⋅0	93⋅0	30⋅0	
Fish oil^g^	22⋅0	80⋅0	160⋅0	0⋅0	48⋅0	112⋅0	
Soya lecithin^h^	10⋅0	10⋅0	10⋅0	10⋅0	10⋅0	10⋅0	
Vitamin and minerals premix^i^	20⋅0	20⋅0	20⋅0	20⋅0	20⋅0	20⋅0	
Astaxanthin^j^	1⋅5	1⋅5	1⋅5	1⋅5	1⋅5	1⋅5	
*Proximate composition (g*/*kg as fed)*							
Moisture	78⋅0	81⋅0	82⋅0	86⋅0	81⋅0	71⋅0	916⋅1
Ash	68⋅1	68⋅0	66⋅2	68⋅70	68⋅2	66⋅3	11⋅6
Crude protein	400⋅0	397⋅0	385⋅0	497⋅0	495⋅0	481⋅0	58⋅2
Total lipid	107⋅0	147⋅0	233⋅0	85⋅8	119⋅0	172⋅0	9⋅6
Carbohydrates^x^	346⋅9	307⋅0	233⋅8	262⋅5	236⋅8	209⋅7	4⋅5
L:CHO ratio	0⋅3	0⋅5	1⋅0	0⋅3	0⋅5	0⋅8	2⋅1
Gross energy (kJ/g)^y^	19⋅0	19⋅8	21⋅6	18⋅7	19⋅5	20⋅8	1⋅8
Protein/Energy (g/MJ)	21⋅0	20⋅1	17⋅8	26⋅5	25⋅4	23⋅1	32⋅6

a Micronorse: 70⋅9 % CP, 8⋅7 % CF, Tromsø Fiskeindustri AS, Norway.

b Squid meal: 83 % CP, 4 % CF, Sopropêche, France.

c Krill meal: 61⋅1 % CP, 17⋅4 % CF, Aker Biomarine, Norway.

d VITAL: 80⋅4 % CP, 5⋅6 % CF, Roquette, France.

e Wheat meal: 11⋅7 % CP, 1⋅6 % CF, Molisur, Spain.

f Meritena 200: 0⋅4 % CP, 0⋅1 % CF, 90 % starch, Tereos, France.

g Fish oil: 98⋅1 % CF, 16 % EPA, 12 % DHA, Sopropêche, France.

h P700IPM, Lecico GmbH, Germany.

i Vitamins (IU or mg/kg diet): DL-alpha tocopherol acetate, 200 mg; sodium menadione bisulphate, 50 mg; retinyl acetate, 40 000 IU; DL-cholecalciferol, 4000 IU; thiamine, 60 mg; riboflavin, 60 mg; pyridoxine, 40 mg; cyanocobalamin, 0⋅2 mg; nicotinic acid, 400 mg; folic acid, 30 mg; ascorbic acid, 1000 mg; inositol, 1000 mg; biotin, 6 mg; calcium pantothenate, 200 mg; choline chloride, 2000 mg, betaine, 1000 mg. Minerals (g or mg/kg diet): copper sulphate, 18 mg; ferric sulphate, 12 mg; potassium iodide, 1 mg; manganese oxide, 20 mg; sodium selenite, 0⋅02 mg; zinc sulphate, 15 mg; sodium chloride, 800 mg; excipient wheat gluten, Premix Lda., Portugal.

j Carophyll Pink 10 % CWS, 10 % astaxanthin, DSM Nutritional Products, Switzerland.

x Estimated by difference: Carbohydrates (%) = 100 − (Crude protein + Total lipid + Ash).

y Gross energy (MJ/kg) = Protein content × 21⋅3 kJ/g + Lipid content × 39⋅5 kJ/g + Carbohydrate content × 17⋅6 kJ/g)/1000 kJ/MJ^(46^^)^.

**Table 2.** Amino acid profile (g per 100 g as is) of experimental extruded feeds and Antarctic krill

Protein level	400 g/kg			500 g/kg			
L:CHO ratio	LOW	MED	HIGH	LOW	MED	HIGH	CTRL
Amino acids (g per 100 g)							
*Essential*							
Arg	2⋅16	2⋅00	2⋅02	2⋅80	2⋅77	2⋅41	2⋅49
His	0⋅74	0⋅66	0⋅65	0⋅98	1⋅02	0⋅84	0⋅91
Ile	1⋅54	1⋅43	1⋅44	1⋅91	1⋅96	1⋅65	1⋅92
Leu	2⋅61	2⋅42	2⋅42	3⋅24	3⋅33	2⋅80	3⋅13
Lys	2⋅29	2⋅01	1⋅99	2⋅69	2⋅78	2⋅30	3⋅41
Met	1⋅15	1⋅09	0⋅87	1⋅44	1⋅51	0⋅99	0⋅76
Phe	1⋅48	1⋅39	1⋅39	1⋅89	1⋅90	1⋅61	1⋅89
Thr	1⋅48	1⋅39	1⋅40	1⋅86	1⋅91	1⋅61	2⋅05
Val	1⋅75	1⋅64	1⋅64	2⋅11	2⋅19	1⋅85	2⋅28
∑EAA	15⋅18	14⋅01	13⋅79	18⋅91	19⋅35	16⋅04	18⋅82
*Nonessential*							
Ala	2⋅01	1⋅85	1⋅84	2⋅40	2⋅48	2⋅10	2⋅88
Asp	3⋅31	3⋅03	3⋅06	3⋅95	4⋅07	3⋅47	4⋅37
Cys	0⋅04	0⋅03	0⋅03	0⋅04	0⋅02	0⋅03	
Glu	5⋅36	4⋅86	4⋅81	6⋅99	7⋅07	5⋅99	5⋅85
Gly	2⋅41	2⋅24	2⋅23	2⋅97	3⋅07	2⋅66	3⋅04
Pro	2⋅17	2⋅02	2⋅02	2⋅76	2⋅89	2⋅49	2⋅63
Ser	1⋅46	1⋅38	1⋅40	1⋅93	1⋅92	1⋅63	1⋅74
Tau	0⋅24	0⋅21	0⋅20	0⋅29	0⋅27	0⋅19	1⋅88
Tyr	1⋅30	1⋅22	1⋅23	1⋅64	1⋅70	1⋅41	1⋅59
∑NEAA	18⋅28	16⋅81	16⋅79	22⋅97	23⋅46	19⋅96	23⋅97
∑TAA	33⋅46	30⋅82	30⋅58	41⋅88	42⋅81	36⋅00	42⋅78

### Growth trial

Prior to the start of the experiment, 24 h unfed lobster juveniles (stage V to VI) were individually weighed and measured (carapace length). Homogeneous groups of 30 individuals (initial weight of 90⋅0 ± 2⋅8 mg per lobster; carapace length of 6⋅5 ± 0⋅6 mm, mean (sd)) were randomly allocated to each dietary treatment. As each lobster was held separately, the experimental unit in the present study was the individual.

Individual lobsters were hand-fed in excess a pre-weighed amount of thawed krill or extruded pellet each morning, and allowed to feed for 4 h. Thus, all lobsters had equal access to feed for a limited amount of time per day. Additionally, lobsters were permitted to consume their shedded exoskeletons. After each meal, the uneaten feed was siphoned off and stored in 50 ml tubes at −20°C. To estimate feed intake (FI), each treatment was divided into subgroups of ten individuals. The uneaten feed fraction of each subgroup (minus sampled or dead animals, when applicable) was collected daily, accumulated over 6 weeks and stored in a single tube, allowing triplicate values to be obtained for FI estimation. At the end of the trial, each tube content was filtered, dried (24 h at 60°C) and weighed for FI estimation using the following formula^(47)^:
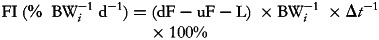
where dF is distributed feed, uF is unconsumed feed, L is leaching after 4 h, BW_i_ is the initial body weight and Δ*t* is the number of days during which uneaten feed was collected (42 d). Leaching was estimated by placing a pre-weighed quantity of each diet in the cassette compartments under the same conditions as in the trial but, in this case, without animals. FI was expressed in the percent of DM ingested per initial body weight per day. Energy intake was calculated by multiplying the daily individual intake (mg) by the gross energy content of each diet.

The presence of shedded exoskeletons and mortality were recorded daily. The experiment was conducted for 8 weeks and lobsters were individually weighed and measured every second week. Significant mortality was gradually observed in the group of animals fed the 400HIGH diet. Consequently, at week 6, samples for enzyme activity (nine individual lobsters per treatment) were collected and the growth trial ended for the 400HIGH group. The growth trial continued for two more weeks for the other groups. Body wet weight was recorded to the nearest 0⋅001 g after gently blotted dry each individual lobster with a paper towel. Carapace length was recorded to the nearest 0⋅1 mm with a Vernier calliper from the base of the eye socket to the posterior edge of the cephalothorax. The following formulas were used to determine growth performance:
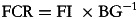
where FCR is the feed conversion ratio; FI is feed intake (dry weight, mg); BG is the biomass gain (wet weight, mg).
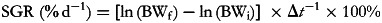
where SGR is the specific growth rate; BW_f_ is the final wet body weight (at week 6); BW_i_ is the initial wet body weight and Δ*t* is the number of growth trial days (42) considered for SGR estimation.
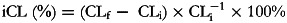
where iCL is the increment in carapace length; CL_f_ is the final carapace length (at 42nd day) and CL_i_ is the initial carapace length.

### Juvenile lobsters proximate chemical composition

At the end of the trial, lobsters were lethally anaesthetised in ice-cold seawater, weighed, measured, rinsed in distilled water and stored at −80°C for proximate chemical composition analysis. Proximate analysis of the juveniles was performed in analytical triplicates per dietary treatment. Briefly, the pool of six to twelve individuals per treatment were finely ground using a Krups Speedy Pro homogenizer and analysed for DM and ash^(43)^. Protein was determined spectrophotometrically at 750 nm using a commercial Lowry-based, micro-protein determination kit (BIO-RAD 500-0112). Lipids were extracted with chloroform–methanol (2:1 by volume) according to the Folch method^(48)^ and lipid content determined gravimetrically.

#### Nucleic acid determinations

At week 4, six juveniles per dietary treatment were collected for nucleic acid analysis 24 h after being fed. Lobsters were lethally anaesthetized in ice-cold seawater, weighed, measured and rinsed in distilled water before being frozen at −80°C. Lobsters were freeze-dried and weighed (±1 μg dry weight (DW)) on an electronic microbalance (Sartorius M5P). The concentration of nucleic acids (RNA, DNA) was quantified in approximately 1 mg of dry abdominal muscle tissue from each individual lobster following procedures described previously^(49)^. Briefly, the muscle samples were chemically (cold sarcosyl Tris-EDTA extraction buffer) and mechanically homogenised in an ultrasonic homogeniser unit (4710 Series, Cole Parmer Instruments Co.). The homogenate was centrifuged for 15 min at 1200*g*, 4°C, and the supernatant extract was used for the analysis of RNA and DNA. The supernatants (30 μl) were placed into fluorescent microplate wells with Tris buffer (140 μl). Finally, a specific nucleic acid fluorochrome dye GelRED (30 μl) was added into each well for the fluorescent reading of nucleic acids. Fluorescence was measured on a microplate reader (Biotek Synergy HT model SIAFRTD – BioTek Instruments, Inc., Vermont, USA) with an excitation wavelength of 365 nm and an emission wavelength of 590 nm. Following the first scan to determine the total fluorescence of RNA and DNA, a ribonuclease A (Type-II A) solution (30 μl) was added to each well and activated by incubating the fluorescent plates at 37°C for 30 min. The plates were read again, and the concentration of DNA was calculated directly from the standard curve described later. The RNA fluorescence was calculated subtracting the DNA fluorescence (second scan) from total fluorescence (first scan). Concentrations were determined by running standard curves of DNA-GelRED and RNA-GelRED with known concentrations of ƛ-phagus DNA (0⋅25 μg/μl) and 16S-23S *E. coli* RNA (4 μg/μl) (Roche). The average ratio of DNA and RNA slopes (average  and  sd) was 5⋅85 ± 0⋅01. The RNA/DNA ratios were standardised (sRD) using DNA and RNA slope ratios and the reference slope ratio of 2⋅4^(50)^. The physiological condition of the juveniles was estimated with nucleic acid derived indices: RNA (μg/mg DW), DNA (μg/mg DW) and sRD (standardised RNA/DNA ratios).

#### Digestive enzyme activities

At week 6, nine lobsters per treatment were collected between 4 h to 5 h after being fed for the analysis of digestive enzyme activities. Individuals were rinsed in distilled water and stored at −80°C. To prepare the enzyme extracts, juveniles were previously freeze-dried. Rostrum, chelipeds, legs, pleopods, uropods and telson were removed and the abdominal section of each individual was mechanically homogenised in 1 ml distilled water. The homogenate was centrifuged for 10 min at 15 800*g*, 4°C, and the supernatant extract was used for the analysis of trypsin, amylase and lipase activities. All samples were kept in ice all times to avoid enzymes denaturation or damage. Enzyme extract aliquots were stored at −80°C until analysis.

For trypsin analysis, the fluorogenic substrate Boc-Gln-Ala-Arg-7-methylcoumarin hydrochloride (BOC-SIGMA B4153) was diluted in dimethyl sulfoxide (DMSO), to a final concentration of 20 μM. For analysis, 5 μl of this substrate, 190 μl of 50 mm Tris + 10 mm CaCl_2_ buffer (pH 8⋅5) and 15 μl of the enzyme extract were added to the microplate^(51)^. Fluorescence was measured at 355 nm (excitation) and 460 nm (emission).

Ultra Amylase Assay Kit (E33651) from Thermo Scientific was used for amylase analysis. The kit contains a starch derivate labelled with a fluorophore dye as a substrate. This substrate was diluted in 3-(*N*-morpholino) propane sulfonic acid (MOPS; pH 6⋅9) and substrate solvent (sodium acetate; pH 4⋅0), to a final concentration of 200 μg/ml. For analysis, 50 μl of the substrate solution and 15 μl of the enzyme extract were added to the microplate. Fluorescence was measured at 485 nm (excitation) and 538 nm (emission).

Lipase activity was assayed using 4-methylumbelliferyl heptanoate (M2514, Sigma-Aldrich). The substrate was dissolved in phosphate buffer pH 7⋅0 to a final concentration of 0⋅4 mm, modified method from Ref. (51). Fifteen microlitres of the lobster extract was added to the microplate and mixed with 250 μl of 0⋅4 mm substrate for the analysis. Fluorescence was measured at 355 nm (excitation) and 460 nm (emission).

All enzyme activities were expressed as RFU (Relative Fluorescence Units) per mg lobster dry weight before dissection.

### Statistical analyses

The amino acid profiles of control diet and experimental feeds were analysed by principal component analysis performed using R version 3.5.1 software^(52)^ and the factoextra version 1.0.7 package^(53)^. Survival and moult occurrence data were analysed by a Kaplan–Meier procedure. Log-rank (Mantel–Cox) test was used to determine significance (*P* < 0⋅05). Whenever significance was detected, a *χ*^2^ table with multiple comparisons was generated to identify differences among dietary treatments. All other parameters shown are expressed as means (sem) unless otherwise specified. Before analyses, the ANOVA assumptions of normality of residuals and homogeneity of variances were tested using the Shapiro–Wilk and Levene's test, respectively. In instances where assumptions were not met, data were square root or log-transformed. A second-order polynomial regression with time was used as an estimate of the average growth, both in terms of carapace length and body weight. The models were validated via residual plot analysis and generated using R version 3.5.1 software^(52)^. Curve coefficients were compared in a one-way ANOVA and whenever significant differences were detected, the coefficients from experimental feed curves were tested against the CTRL using the Dunnett *t*-test. For the remaining estimated growth parameters, RNA and DNA concentrations, sRD and digestive enzyme activities, the dietary treatments were subjected to a one-way ANOVA to test the experimental formulated dry feeds against the CTRL diet. Whenever significant differences were identified, comparisons against the CTRL diet were conducted using the Dunnett *t*-test. Data from experimental extruded feed treatments were subsequently subjected to a two-way ANOVA, considering the protein level and the L:CHO ratio as explanatory variables. Following the two-way ANOVA and, whenever significant differences were identified, means were compared by the Holm-Sidak *post hoc* test. A standardised RNA/DNA ratio was expected to reflect changes in growth performance parameters^(54)^. Thus, a simple linear regression was calculated to predict sRD based on SGR. Apart from the principal component analysis and the second-order polynomial regression for carapace length and body weight, all statistical tests were performed using the IBM SPSS Statistics 25.0 and graphics were generated by GraphPad Prism version 5.0 software package.

## Results

### Diets amino acid composition

**Fig. 1.**
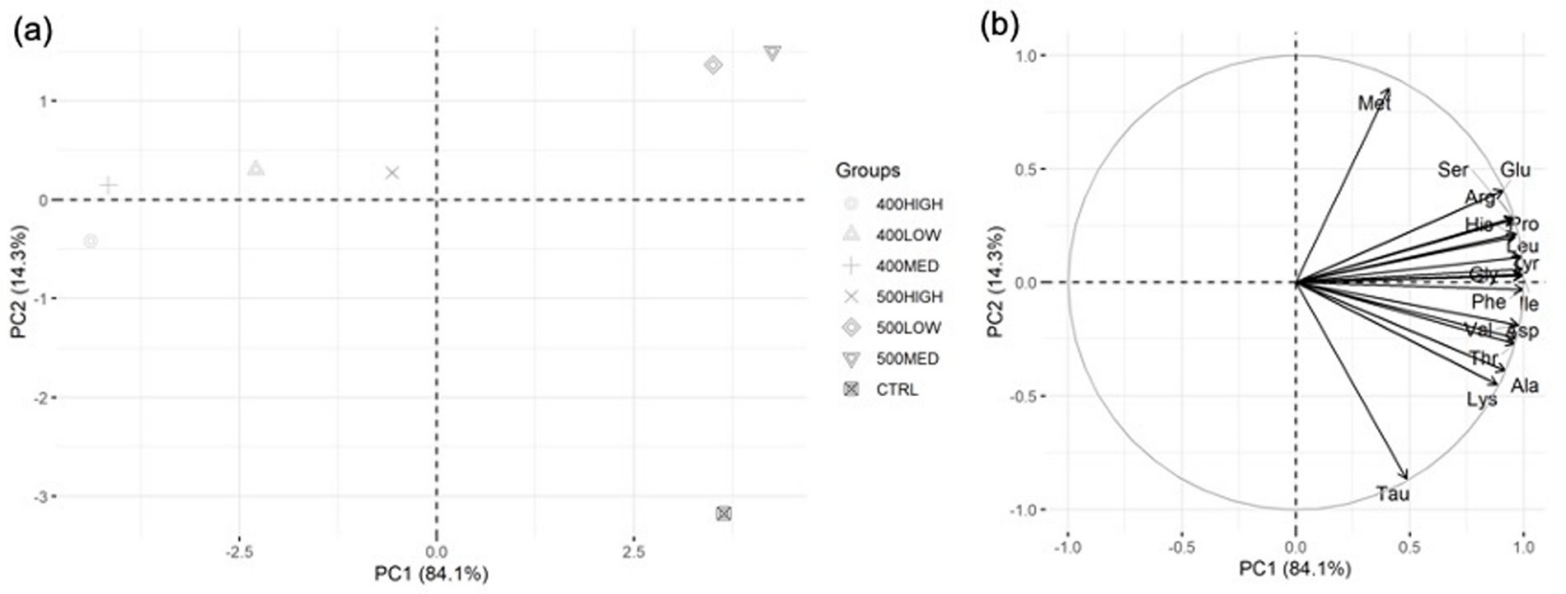
Graphical representation of principal components analysis of amino acid profiles from tested diets. The two panels are complementary to one another. (a) Biplot of the first two principal components (PC1 and PC2) of tested diets amino acid profiles. PC1 separated the amino acid profiles horizontally and explained 84⋅1 % of the variance. The amino acid profiles of 500MED, 500LOW and CTRL diets formed a succinct group to the right of the plot and were positively correlated to PC1. The 400HIGH, 400MED, 400LOW and 500HIGH diets were negatively correlated to PC1. PC2 separated the amino acid profiles vertically and explained 14⋅3 % of the variance. The 500LOW and 500MED diets were positively correlated, while the CTRL was negatively correlated to PC2. (b) Variables (amino acids) used to construct the principal components. The circle in this plot is the correlation circle, the stronger the correlation of an amino acid to PC1 or PC2 the closer its arrowhead to the circle. The arrows indicate how the amino acids contributed to the formation of PC1 and PC2 and thus the formation of plot (a).

The CTRL and 500MED feed contained higher levels of both essential (EAA) and nonessential (NEAA) amino acids, while the 400MED and 400HIGH contained the lowest levels for EAA and NEAA (Table 2). A principal component analysis (Fig. 1) revealed that the 500LOW and 500MED were more similar to the CTRL than any other experimental feeds. The majority of the variance was explained by PC1 (84⋅1 %) which mostly identified differences related to the concentration of the total amino acids that was lower in all the three 400 and in the 500HIGH compared with the other groups. PC2 explained 14⋅3 % of the variability and showed that methionine and taurine were the amino acids with higher contribution for differences between the CTRL and the extruded 500MED and 500LOW feeds.

### Survival, growth and feed utilisation

**Fig. 2.**
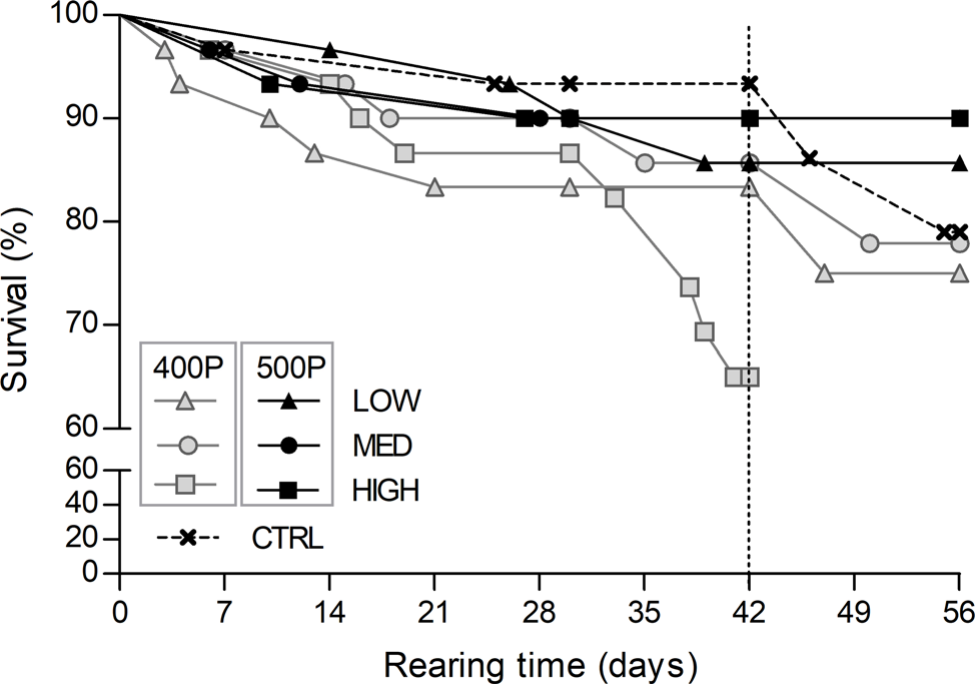
Survival of *H. gammarus* juveniles (% of initial numbers) fed on the different diets. The dashed vertical line indicates the rearing time limit (42 d) considered in the statistical comparison of the curves.

Survival was not significantly affected by dietary treatment (*χ*^2^ 8⋅2, df 6, *P* = 0⋅22) varying between 65 and 90 % (Fig. 2). Although no statistically significant difference was detected in the survival rate of the 400HIGH group, the projected curve indicates a marked divergence from the other dietary groups. In order to secure enough sample size for subsequent analysis, we decided to end the growth trial for this treatment 2 weeks earlier. Therefore, despite the survival curves were calculated for an 8-week period in all treatments other than the 400HIGH, their comparison was performed only for the first 6-week period (Fig. 2).

**Fig. 3.**
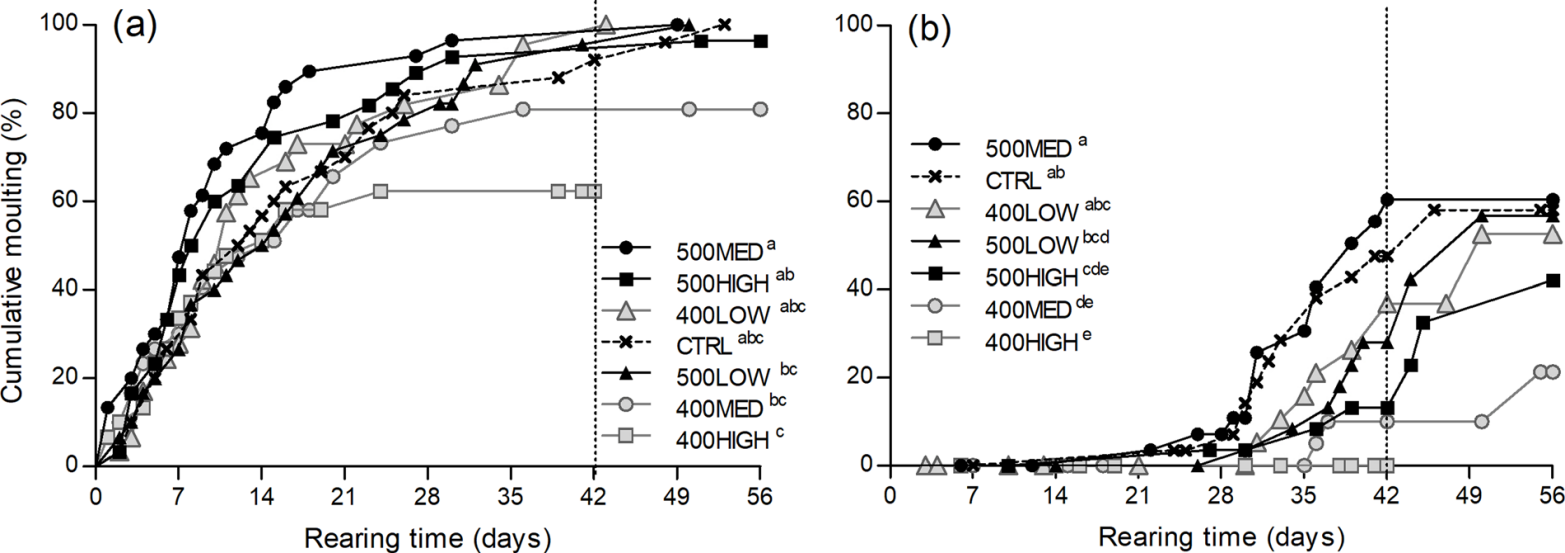
Cumulative moults of *H. gammarus* juveniles (% of initial numbers) fed on different diets. Panel (a) refers to the first moult occurred after the beginning of the growth trial and panel (b) refers to the second moult occurrence. Dietary treatments are indicated in the legend in ascending order of cumulative moults. The dashed vertical line indicates the rearing time limit (42 d) considered in the statistical comparison of the curves. Different superscript letters indicate significant differences between dietary treatments.

As for survival, moulting curves comparison was restricted to the first 6 weeks. Moulting occurrence was significantly affected by dietary treatment (Fig. 3) and the rate of a first moult varied between 62 and 100 % among diets. Mean cumulative first moult was significantly higher for lobsters offered the 500MED feed compared with juveniles fed the 400HIGH, 400MED and 500LOW feeds (*χ*^2^ 14⋅4, df 6, *P* = 0⋅03). Juvenile lobsters offered the 400HIGH diet did not succeed a second moult. In the remaining groups, the occurrence rate of the second moult varied between 21 and 60 %. Mean cumulative successful second moult was significantly higher for the 500MED than the other groups, except for CTRL and 400LOW (*χ*^2^ 32⋅3, df 6, *P* < 0⋅001).

**Fig. 4.**
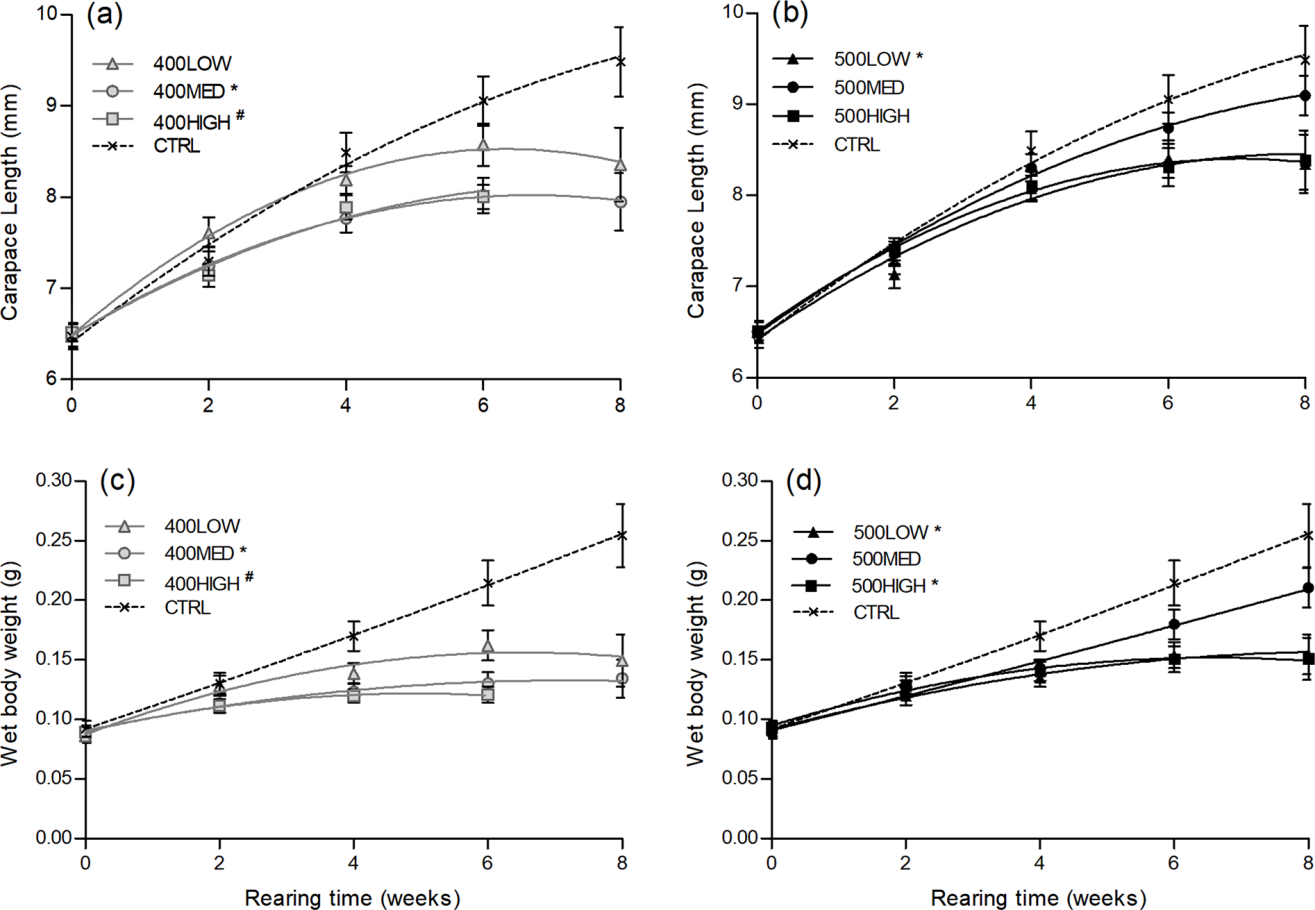
Second-order polynomial model fit to average carapace length (upper panels (a) and (b)) and wet body weight (lower panels (c) and (d)) of *H. gammarus* juveniles fed on different diets throughout the growth trial period. Data points represented as mean (sem). Dietary treatments that were significantly different from CTRL are marked with an asterisk (*). # indicates that curve coefficients from the 400HIGH group were not statistically compared with CTRL.

Changes in weight and carapace length throughout the trial were evaluated by second-order polynomial regression with time. Regression curves are represented for all dietary treatments shown in Fig. 4. However, because the trial ended at week 6 for the 400HIGH treatment, the curve coefficients from this group were not statistically compared with the CTRL. During the 8-week experimental period, lobsters grew from an initial mean weight of 90 mg (6⋅5 mm carapace length) to mean weights ranging from 134 to 254 mg (7⋅9–9⋅5 mm carapace length). The juveniles fed the 500MED and CTRL diets sustained an increase in carapace length and body weight over the 8-week growth trial. The remaining groups reached a growth plateau, both in terms of length and weight, after 6 weeks (Fig. 4). The 400MED and 500LOW experimental feeds had a significant lower carapace length increase over time compared with the CTRL (Fig. 4(a) and 4(b)). In terms of body weight gain over the trial period, all the experimental dietary groups except the 500MED and 400LOW showed a significantly slower growth compared with the reference group (CTRL) (Fig. 4(c) and 4(d)).

The growth performance indices – SGR and iCL – were determined for a 6-week period in all treatments (Table 3). A significantly higher SGR was observed for krill-fed lobsters in comparison with lobsters fed the extruded feeds, except for the 500MED diet (Table 3). Within the extruded feeds, SGR was significantly affected by the protein level, the L:CHO ratio and the interaction of protein × L:CHO ratio. Results showed that for the 400-protein group, juveniles fed the 400LOW diet grew faster than the ones fed the 400MED and 400HIGH feeds. Within the 500-protein level, a significantly faster growth was observed in the 500MED treatment. The protein content did not affect the SGR in the LOW L:CHO ratio category, while in the MED and HIGH groups, juveniles fed the 500 g/kg protein feeds grew significantly faster (Table 3). Juveniles fed the 400MED, 400HIGH and 500HIGH feeds had a significantly lower iCL than the CTRL group. The protein content, L:CHO ratio and protein × L:CHO all had a significant effect on the iCL. Among the 400-protein level, the highest iCL was achieved for the 400LOW and the lowest for the 400HIGH group. Within the 500 level, the highest iCL was observed for the 500MED and the lowest for the 500HIGH treatment. The protein content only had a significant effect on iCL at the intermediate level of L:CHO for which the 500MED were larger than the 400MED group (Table 3).

**Table 3.** Growth performance, feed efficiency and whole body composition of juvenile *H. gammarus* fed the experimental extruded feeds and Antarctic krill

Protein level	400 g/kg			500 g/kg				One-way ANOVA	Two-way ANOVA		
L:CHO ratio	LOW	MED	HIGH	LOW	MED	HIGH	CTRL		P	L:CHO	P × L:CHO
SGR (% d^−1^)	1⋅28 ± 0⋅13^#,ax^	0⋅77 ± 0⋅09^#,bx^	0⋅50 ± 0⋅11^#,bx^	1⋅29 ± 0⋅13^#,Bx^	1⋅72 ± 0⋅13^Ay^	1⋅06 ± 0⋅07^#,By^	1⋅89 ± 0⋅13	*F*_6,137_ 17⋅10***
*F*_1,115_ 29⋅17***	*F*_2,115_ 11⋅29***	*F*_2,115_ 8⋅86***
iCL (% CL_i_)	29⋅0 ± 2⋅3^ax^	21⋅2 ± 2⋅4^#,abx^	19⋅3 ± 2⋅8^#,bx^	30⋅4 ± 2⋅2^ABx^	35⋅2 ± 2⋅5^Ay^	25⋅4 ± 1⋅6^#,Bx^	37⋅5 ± 2⋅7	*F*_6,137_ 8⋅04***	*F*_1,115_ 14⋅63***	*F*_2,115_ 5⋅60**	*F*_2,115_ 4⋅00*
*N*	19	20	15	20	21	21	22				
FI (%BW_i_ d^−1^)	10⋅1 ± 0⋅6^#,ax^	4⋅6 ± 0⋅8^#,bx^	2⋅7 ± 0⋅2^cx^	7⋅3 ± 0⋅3^#,Ay^	5⋅3 ± 0⋅2^#,Bx^	5⋅1 ± 0⋅3^#,By^	2⋅7 ± 0⋅2	*F*_6,20_ 34⋅74***	*F*_1,17_ 1⋅59	*F*_2,17_ 53⋅11***	*F*_2,17_ 15⋅64***
EI (J/d)	179⋅2 ± 8⋅8^#,ax^	89⋅1 ± 16⋅3^#,bx^	56⋅6 ± 3⋅9 ^bx^	135⋅7 ± 5⋅5^#,Ay^	100⋅4 ± 3⋅8^#,By^	106⋅6 ± 6⋅2^#,ABy^	53⋅3 ± 4⋅7	*F*_6,20_ 29⋅37***	*F*_1,17_ 0⋅72	*F*_2,17_. 44⋅52***	
FCR	4⋅3 ± 0⋅1^#^	3⋅9 ± 0⋅4^#^	3⋅3 ± 0⋅5^#^	4⋅2 ± 0⋅7^#^	2⋅0 ± 0⋅2	3⋅2 ± 0⋅3^#^	0⋅7 ± 0⋅1	*F*_6,20_ 10⋅45***	*F*_1,17_ 4⋅15	*F*_2,17_ 4⋅94*	*F*_2,17_ 2⋅77
*N*	3	3	3	3	3	3	3				
*Proximate composition (% of DM)*											
Dry matter	24⋅5 ± 0⋅4	22⋅7 ± 1⋅1	19⋅9 ± 1⋅2	25⋅3 ± 0⋅4	23⋅7 ± 1⋅8	24⋅2 ± 0⋅6	29⋅1 ± 0⋅5				
Ash	34⋅7 ± 0⋅6	36⋅8 ± 0⋅3	42⋅8 ± 1⋅1	35⋅0 ± 0⋅4	34⋅0 ± 0⋅3	34⋅7 ± 0⋅5	32⋅3 ± 0⋅5				
Soluble protein	50⋅4 ± 3⋅1	47⋅8 ± 4⋅1	42⋅2 ± 1⋅8	40⋅6 ± 1⋅4	42⋅9 ± 2⋅4	49⋅8 ± 3⋅4	45⋅4 ± 1⋅7				
Total lipid	6⋅3 ± 1⋅3	6⋅4 ± 0⋅4	3⋅5 ± 0⋅8	7⋅0 ± 0⋅7	5⋅5 ± 1⋅0	5⋅2 ± 0⋅2	5⋅4 ± 1⋅1				
Carbohydrates^†^	8⋅6 ± 3⋅1	9⋅0 ± 4⋅0	11⋅5 ± 0⋅7	17⋅5 ± 2⋅3	17⋅6 ± 1⋅6	10⋅4 ± 3⋅9	17⋅0 ± 2⋅9				

SGR, specific growth rate; iCL, increment in carapace length; FI, dry feed intake; FCR, dry feed intake/wet weight gain; EI, energy intake, N, number of replicates per treatment.

Values are means (sem).

^#^indicates dietary groups significantly different from CTRL (Krill).

Means in the same row with a different superscript ‘a, b, c’ or ‘A, B, C’ are significantly different within the 400- or 500-protein level, respectively.

Means in the same row with a different superscript ‘x or y’ are significantly different within the same L:CHO ratio category.

† Estimated by difference: Carbohydrates (%) = 100 − (Soluble protein + Total lipid + Ash).

* *P* < 0⋅05, **P < 0⋅01, ***P < 0⋅001.

As for SGR and iCL, the feed efficiency indices – energy intake, FI and FCR – were calculated for the first 6-week period in all treatments (Table 3). FI was significantly higher for all experimental feeds compared with the CTRL except for the 400HIGH. Within the experimental feeds, the L:CHO ratio and the interaction of protein × L:CHO had a significant effect on the FI. A higher FI in extruded feeds with a higher carbohydrate content was observed for both protein levels examined (400 and 500 mg/kg) with most pronounced differences in feeds with a lower protein content. As such, lobsters fed LOW feeds presented higher FI than HIGH with intermediate levels observed in the MED treatments (Table 3). Intake expressed in terms of energy followed the same trend as FI. The FCR was significantly lower for the CTRL diet compared with the experimental extruded feeds except for the 500MED group. There was a significant effect on FCR from the L:CHO ratio among the experimental dry feeds, for which FCR in juveniles reared on MED feeds was significant lower compared with LOW feeds (Table 3).

### Proximate composition

No statistical analysis was performed on the proximate composition data set since only analytical replicates were used in the analysis of a single pooled sample. The DM content varied from 20 to 29 %, with a minimum observed for the 400HIGH and maximum for the CTRL group. The inverse trend was detected for the ash content, with a minimum of 32 % observed for the CTRL and a maximum of 43 % for the 400HIGH treatment. The protein fluctuated between 41 and 50 %, lipids between 4 and 7 % and carbohydrates between 9 and 18 %. Juveniles reared on the 500LOW experimental diet had the highest lipid and the lowest protein content, while the lobsters fed the 400LOW diet had the highest protein and lowest carbohydrate contents. Minimum lipid content was observed for the 400HIGH group and maximum carbohydrate content for the 500MED group (Table 3).

#### Nucleic acid derived indices

**Fig. 5.**
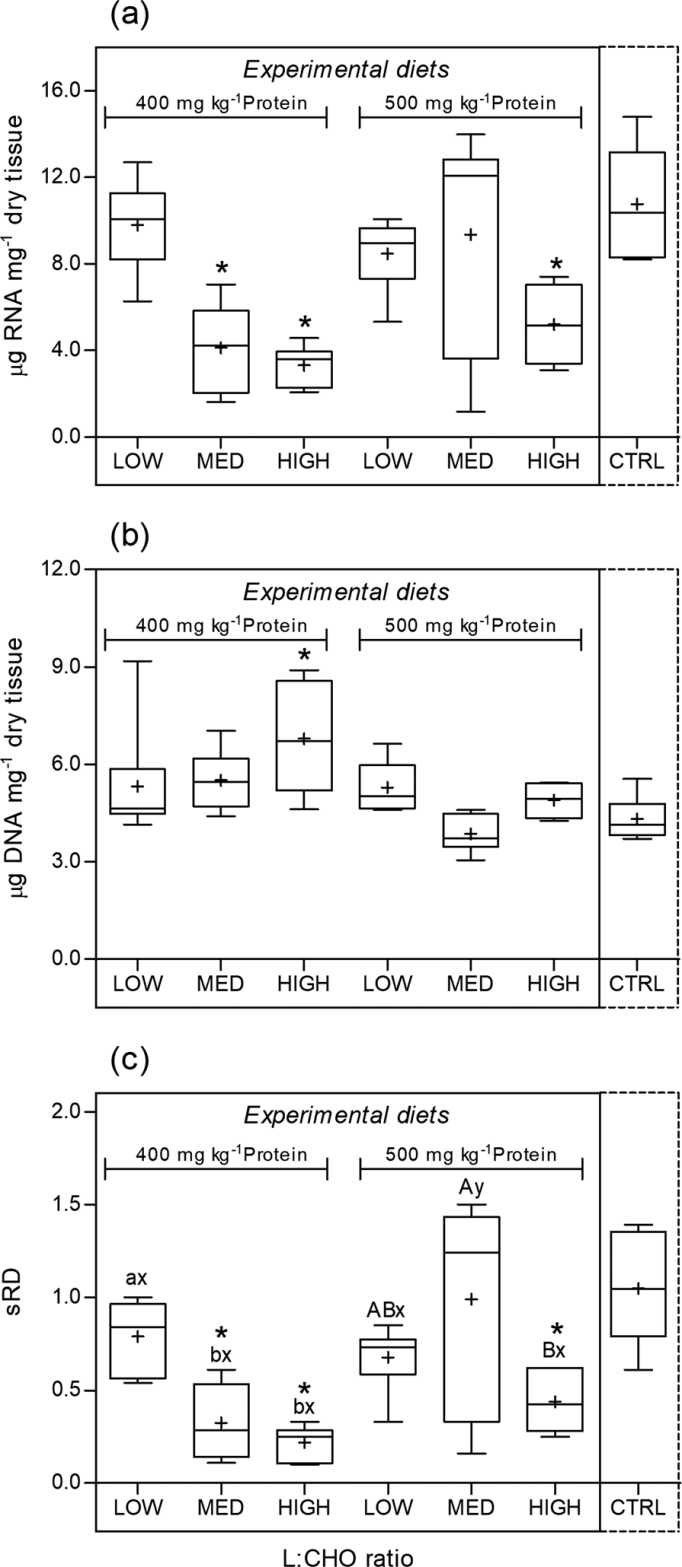
Changes in RNA concentration (a), DNA concentration (b) and standardised RNA/DNA ratio, sRD (c) of abdominal muscle tissue of *H. gammarus* juveniles fed on different diets (*n* 6). The box includes observations from the 25th to 75th percentiles, and the whiskers above and below the box indicate the 10th and 90th percentiles. The horizontal line within the box represents the median value and the symbol (+) indicates the mean. Dietary treatments that were significantly different from CTRL are marked with an asterisk (*). Different letters ‘a, b’ or ‘A, B’ indicate significant differences within the 400- or 500-protein level, respectively. Different ‘x or y’ indicate significant differences within the same L:CHO ratio category.

The changes in the weight-specific RNA and DNA concentrations and sRD are shown in Fig. 5. The RNA content was significantly lower (*F*_6,41_ 5⋅61, *P* < 0⋅001) in animals fed the 400MED, 400HIGH and 500HIGH feeds when compared with the krill-fed lobsters. Within the experimental feeds, the RNA content was affected by the L:CHO ratio (*F*_2,35_ 7⋅61, *P* < 0⋅002). Feeds in the HIGH category triggered a significant reduction in the RNA content of the abdominal muscle (Fig. 5(a)).

In comparison with the CTRL group, the 400HIGH treatment presented a significant increase (*F*_6,41_ 5⋅08, *P* = 0⋅001) in DNA content. Among the experimental extruded feeds, both protein (*F*_1,35_ 10⋅41, *P* = 0⋅003) and L:CHO ratio (*F*_2,35_ 3⋅75, *P* = 0⋅035) significantly affected the DNA concentration in the muscle. Feeds within the 400-protein group supported a significant increase in DNA content compared with the 500-protein group. The DNA content of the abdominal muscle of juveniles reared on the L:CHO MED category feeds was significantly lower compared with the HIGH groups (Fig. 5(b)).

**Fig. 6.**
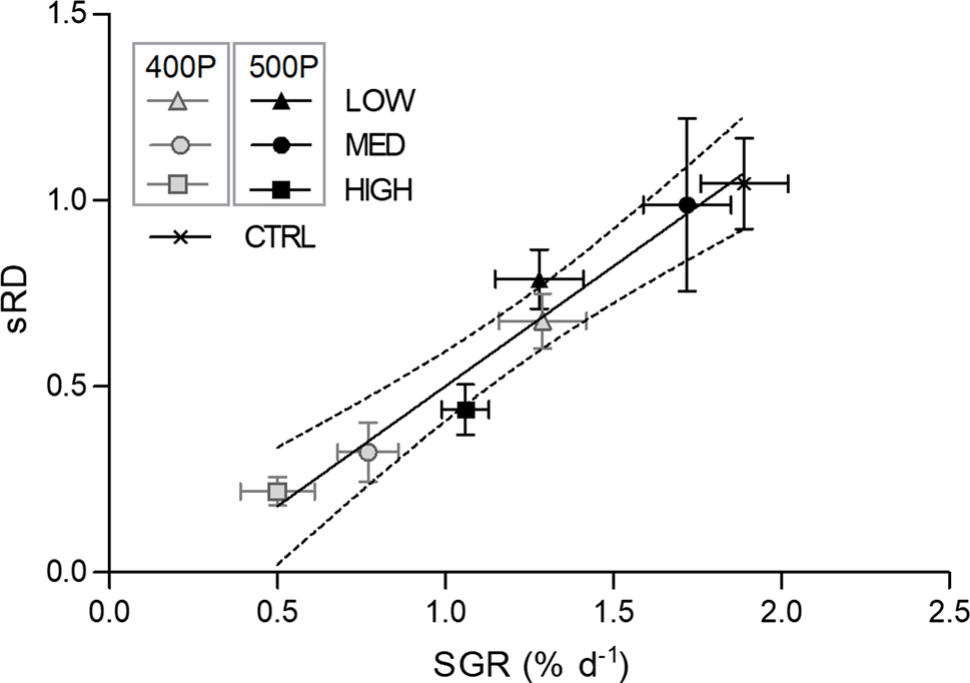
Fitted linear regression model of standardised RNA/DNA ratio (sRD) vs. specific growth rate (SGR). Data points per dietary treatment represented as mean (sem). The solid line displays the average estimates of the predicted sRD and the dashed lines display the 95 % confidence limits. Simple linear regression model: *y*  = 0.644*x* − 0.143 (*R*^2^  = 0.55).

The standardised ratio (sRD) was significantly affected by dietary treatment. The sRD was significantly lower (*F*_6,41_ 7⋅87, *P* < 0⋅001) in animals fed the 400MED, 400HIGH and 500HIGH feeds when compared with the CTRL. Within the experimental feeds, sRD was significantly affected by protein (*F*_1,35_ 7⋅63, *P* = 0⋅010), L:CHO ratio (*F*_2,35_ 7⋅09, *P* = 0⋅003) and the interaction of protein × L:CHO (*F*_2,35_ 5⋅83, *P* = 0⋅007). For the 400-protein level, juveniles fed the 400MED and 400HIGH feeds showed a significantly lower ratio compared with the lobsters fed the 400LOW diet. For the 500 level, the 500MED showed higher sRD than the 500HIGH group. Protein level had a significant effect within the MED L:CHO category, for which the 500 resulted in a higher sRD than the 400 group (Fig. 5(c)). A significant regression equation was found between sRD and SGR (*F*_1,40_ 49⋅05, *P* < 0⋅001), with an *R*^2^ of 0⋅55. The predicted sRD for juvenile lobsters was equal to − 0.143  + 0.644 (SGR) when SGR is estimated in % d^−1^ (Fig. 6).

### Digestive enzyme activities

**Fig. 7.**
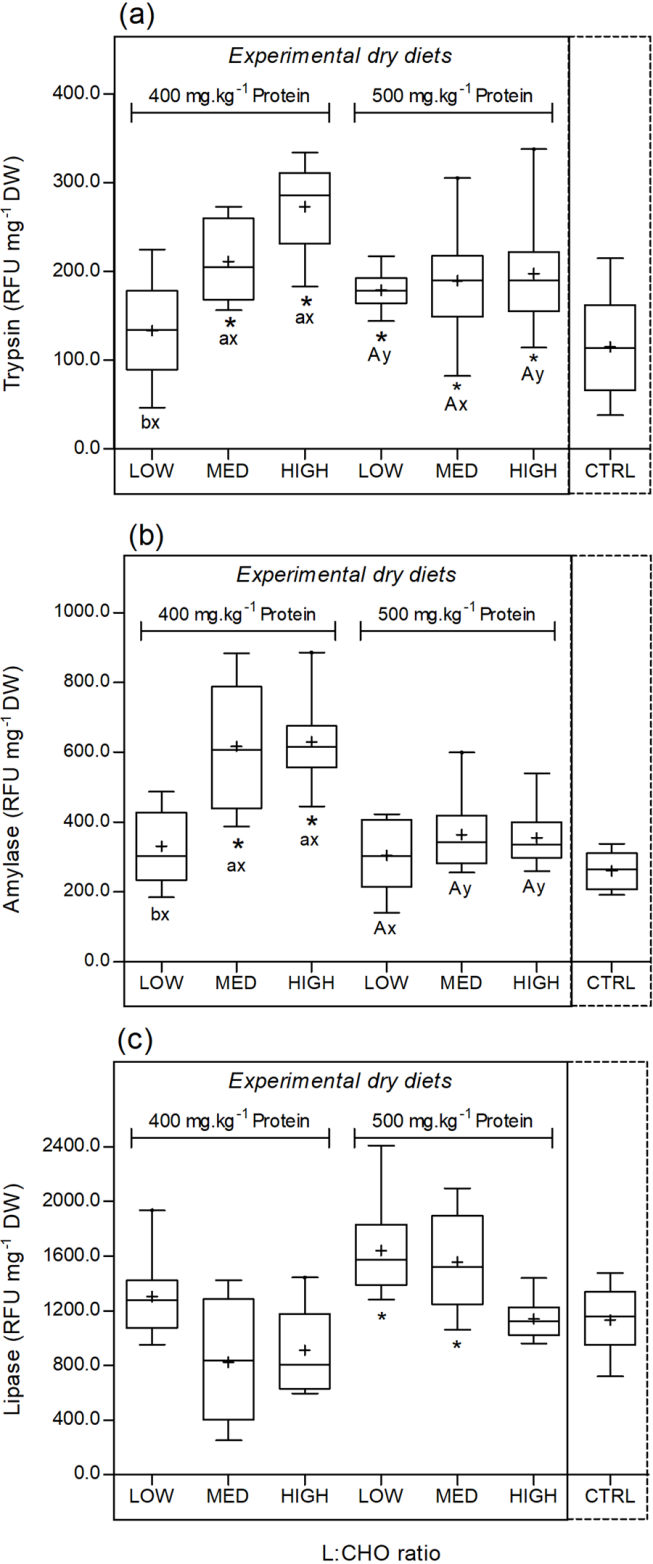
Changes in trypsin (a), amylase (b) and lipase (c) activity of *H. gammarus* juveniles fed on the different tested diets (*n* 9). The box includes observations from the 25th to 75th percentiles, and the whiskers above and below the box indicate the 10th and 90th percentiles. The horizontal line within the box represents the median value and the symbol (+) indicates the mean. Dietary treatments that were significantly different from CTRL are marked with an asterisk (*). Different letters ‘a, b’ or ‘A, B’ indicate significant differences within the 400- or 500-protein level, respectively. Different ‘x or y’ indicate significant differences within the same L:CHO ratio category.

Trypsin activity was significantly higher for the animals fed the experimental feeds than for the CTRL group, except for the 400LOW treatment (*F*_6,62_ 8⋅37, *P* < 0⋅001). Within the experimental feeds, trypsin activity was affected by the L:CHO ratio (*F*_2,53_ 9⋅86, *P* < 0⋅001) and the interaction of protein × L:CHO (*F*_2,53_ 6⋅49, *P* = 0⋅003). No differences were observed among the three feeds in the 500-protein level, but in the 400 group, the 400HIGH and 400MED treatments promoted a significant increase in trypsin activity compared with the 400LOW. The dietary protein content significantly affected the trypsin activity for the LOW (higher for the 500 level) and HIGH (higher for the 400 level) categories (Fig. 7(a)).

As for trypsin, a similar trend was observed for the activity of amylase. When compared with the CTRL, amylase activity was significantly higher in the 400MED and 400HIGH treatments (*F*_6,62_ 5⋅00, *P* < 0⋅001). Protein (*F*_1,53_ 28⋅91, *P* < 0⋅001), L:CHO ratio (*F*_2,53_ 12⋅79, *P* < 0⋅001) and the interaction of both factors (*F*_2,53_ 4⋅97, *P* = 0⋅011) had a significant effect on amylase activity. No significant differences were observed within the 500-protein level. For the 400-protein group, amylase activity was significantly higher in juveniles fed the 400MED and 400HIGH feeds. Within the MED and HIGH categories, amylase activity increased for the 400-protein level (Fig. 7(b)).

Juveniles fed the 500LOW and 500MED feeds had a higher lipase activity in comparison with juveniles fed the CTRL diet (*F*_6,62_ 8⋅33, *P* < 0⋅001). Within the experimental dietary groups, both the protein level (*F*_1,53_ 23⋅36, *P* < 0⋅001) and L:CHO ratio (*F*_2,53_ 8⋅39, *P* = 0⋅001) significantly affected the lipase activity. The activity of this enzyme was significantly higher for the 500-protein content feeds. Within the L:CHO ratio, juveniles fed the LOW feeds had a significantly higher lipase activity than the ones fed the MED and HIGH feeds (Fig. 7(c)).

## Discussion

### Growth performance

Results obtained indicate that the overall performance of European lobster juveniles reared on the 500MED extruded feed for an 8-week period was comparable to the performance of juveniles fed thawed Antarctic krill (CTRL). Previous nutritional studies carried out on *H. gammarus*^(28)^, *H. americanus*^(27)^ and several Palinuridae species^(22)^ showed that the growth rate was generally poorer in lobsters fed formulated compared with fresh diets. A recent study^(18)^ investigated the effect of the same feeds that are used here on the performance, metabolic rate and nitrogen retention in European lobster juveniles. The results indicated that *H. gammarus* grew faster when fed the 500 g/kg compared with the 400 g/kg protein feeds. The results also suggested a potential protein sparring capacity in the formulated feeds, when compensated by the inclusion of carbohydrates, given that the growth obtained for the 400LOW was comparable to that of the three 500 feeds. In the present study, we found that the growth rate of juveniles fed the 400LOW, 500LOW and 500HIGH feeds was lower than previously projected. This is most likely due to the too short growth trial period in the previous study. We, thus, confirm what had already been hypothesised in the previous work, i.e., a period of 4 weeks is not long enough to assess the effects of experimental feeds on the growth of juvenile European lobsters. Even the present study is arguably a little too short. As growth in crustaceans, as opposed to fish, rely on sequential moultings^(55)^, nutritional trials performed in crustacean species aiming growth performance as the primary outcome must consider a minimum duration that allows individuals to complete two moults.

The poorest overall performance observed in the group of lobsters reared on the 400HIGH diet is most likely related to the low FI. The reason for such low intake in this dietary group remains unclear, but it might be associated with a lower palatability, different smell or that the higher lipid content caused faster satiation. Results from a feeding trial carried out with European lobster juveniles of similar size^(56)^ showed that animals fed 5 % dry feed of body mass grew significantly less than juveniles fed 10 %. In the present study, only lobsters fed the 400LOW diet were close to the 10 % level recommended^(56)^, but a direct comparison between the current and the mentioned study is not possible since both test diets and feeding duration were different. In the cited study^(56)^, juveniles were allowed to feed from the afternoon until the next morning, while only 4 h in our set-up. Nonetheless, the fact that low intake compromised growth was validated in both studies. In animals fed the 400HIGH diet, not only growth but also survival was compromised. Results suggest that juveniles were not taking enough feed to support growth, nor even enough to sustain the minimum standard metabolic rate (SMR). In our previous study using the same feeds^(18)^, a significant decrease was observed in the SMR of European lobster juveniles reared on the 400MED and 400HIGH feeds for a 32-d period. Another likely reason for such a low performance on this diet may be related to a low tolerance to high lipid diets by crustaceans^(57)^. It has been previously suggested^(58)^ that high dietary lipids reduce the capacity for amino acid absorption due to an increase in bile acid concentration in response to a high-fat diet.

### Feed efficiency

Although the CTRL group grew faster with the lowest FI among all treatments, the low FI observed for this group is masked by the fact that calculations were performed on a dry weight basis. The CTRL diet composed of frozen Antarctic krill had a much lower DM content (approx. 11 %) than the experimental extruded feeds (DM approx. 90 %) which complicates FI comparisons. Also, the energy intake was lower in lobsters fed with krill compared with the extruded feeds. Moreover, the form in which krill is presented is so different from an extruded feed, which may cause differences in the overall nutrient absorption and digestibility. The highest intake observed for the experimental feeds in the LOW L:CHO category suggests that feeds with the high carbohydrate content are more attractive to European lobster juveniles. This is supported by previous studies showing that the composition of the preferred prey of spiny lobster is generally high in protein content, moderate to high in carbohydrate content and low in lipid content^(23)^. In the present study, we observed that the CTRL group was the most efficient in terms of FCR but highly influenced by the low DM content of the thawed Antarctic krill. When FCR is estimated on a wet weight basis, the trend inverts, for example, FCR increases from 2⋅0 to 2⋅2 and from 0⋅7 to 6⋅0 in the 500MED and CTRL groups, respectively. More meaningful is the comparison of FCR values among the experimental extruded feeds. Results indicate a lower FCR in diet 500MED and points to a better utilisation of this diet. The general best performance of animals fed the 500MED when compared with the other extruded feeds could also be related to a more balanced amino acid composition. This diet, together with the 500LOW, showed the most similarity to the Antarctic krill among the experimental extruded feeds. Still, the apparent lower level of taurine in all the extruded feeds might have limited the growth of juveniles. This amino acid is known to elicit a feed-attractant stimulus in crustaceans^(22)^. A potential solution to increase the performance of lobsters fed on artificial feeds could be the incorporation of taurine to formulated feeds to increase FI of lobsters^(16)^. The 500 mg/kg protein level in the experimental feeds was achieved by increasing the proportion of squid meal. This ingredient is often used in crustaceans’ diets to increase growth and attractiveness of artificial feeds and the replacement of fish by squid meal has been proven beneficial for growth in several crustacean species^(59)^. However, since the amino acid profiles of fish and squid meal are similar^(57)^, it is unlikely that the dissimilarities observed among the experimental extruded feeds were caused by the different inclusion levels of this ingredient.

### Nucleic acid derived indices

The decrease in sRD for the 400MED and 500HIGH dietary groups were mainly caused by a reduction in the RNA content, while for the 400HIGH group, results show that the low sRD was a combined effect of decreased RNA and increased DNA concentrations in the muscle tissue.

The quantified RNA includes ribosomal RNA (rRNA), messenger RNA (mRNA) and transfer RNA (tRNA) which respond in different ways and have different functions. However, since rRNA – responsible for building protein – makes up the majority of total RNA, changes in total RNA were assumed to primarily reflect changes in rRNA^(60)^. Thus, it was presumed that the reduction in RNA reflected a decrease in protein synthesis, while the relative increase in DNA was associated with an increase in the number of cells per tissue portion, however, of smaller dimensions^(61)^. Thus, the results suggest that juveniles reared on the 400HIGH diet were not only affected by a decreased capacity for protein synthesis but also by the mobilisation of proteins from the abdominal muscle to obtain energy, as previously suggested in starving fish larvae^(62)^. Overall, results showed that the sRD was positively correlated with growth performance and feed efficiency indicators. Specifically, we demonstrated that sRD estimated from samples collected at week 4 were significantly correlated with SGR calculated at week 6. These observations suggest that sRD is a sensitive indicator of the nutritional condition of European lobster juveniles. The use of this estimate in crustaceans is of great applicability as it allows the assessment of recent growth and nutritional condition^(33,63,64)^. Most of the nutritional studies performed on crustaceans considered growth rate as gains in wet weight and length. However, the growth process in crustacean species has an interrupted character because is connected to the moult cycle, whereas somatic growth and the accumulation of energy reserves in tissues are a continuous process^(47)^. We conclude that the nucleic derived indices are a useful tool in future nutritional studies with European lobster juveniles as it allows a faster evaluation when compared with conventional growth performance estimators.

### Digestive capacity

The lowest activity levels of trypsin and amylase enzymes were detected in European lobster juveniles reared on Antarctic krill while activities increased in animals offered the 400HIGH and 400MED extruded feeds. This is in contradiction to what was previously reported^(39)^. In that study, was observed that spiny lobsters (*Jasus edwardsii*) reared on a formulated diet had a marked decrease in trypsin and α-amylase activities of the foregut and digestive gland compared with juveniles fed on fresh mussels. A main difference, however, may be that in the present study, enzymatic activity was estimated from the entire abdominal section and not from the foregut and/or hepatopancreas. Thus, the activity detected in tissue homogenates may not necessarily represent the activity of enzymes that will be secreted into the digestive lumen. In crustaceans, proteases and carbohydrases activity was found in tissues outside the gut^(65,66)^. It may also be that the secretion of large amounts of enzymes may maximise the use of limiting nutrients. Such elevated enzymatic activity could maximise hydrolysis and the resulting extraction of a dietary substrate that was ingested in small amounts^(67)^. Results from^(68)^ point to the same hypothesis. In the cited study, the authors reported a significant increase in the specific activity of α-amylase in *J. edwardsii* juveniles fed diets with a low starch inclusion level. Thus, based on this, the results suggest that trypsin and amylase activities increased to compensate for the low levels of protein and carbohydrates lobsters could obtain when fed on the 400MED and 400HIGH feeds. For lipase, the activity was lower for juveniles fed the 400HIGH and 400MED than for individuals reared on the 500LOW and 500MED extruded feeds. While carbohydrates are the primary source of energy for crustaceans^(69)^, lipids are the main energy reserve. European lobsters store the metabolised lipids in the R-cells of the hepatopancreas^(70)^. Overall, the results point toward a combined effect of low protein, high lipid and low FI in the activity of trypsin and amylase. The increased activity of these enzymes in the group of animals fed the 400MED and 400HIGH feeds suggests the activation of a mechanism to optimise the use of protein and carbohydrates as they seem to be the most important nutrients for European lobster juveniles. In contrast, lipase activity increased with protein level in the diet, with significantly higher activity for the 500-protein lobster groups than the 400-protein groups. This suggests that the required supply of protein for growth is covered for the 500 group and that energy reserves to support growth potential are ensured for the lobsters reared on the 500LOW and 500MED feeds.

## Concluding remarks

In the present study, we demonstrated that European lobster juveniles fed the 500MED experimental diet achieved a growth performance and nutritional condition statistically comparable with that observed when animals were fed thawed Antarctic krill. This extruded feed contained 500 g/kg protein, 237 g/kg carbohydrates and 119 g/kg lipids (as fed basis). The results suggest that this composition is the most balanced to meet the overall nutritional requirements of the European lobster juveniles among the tested extruded feeds. Results also support what was previously observed for spiny lobster species: formulated feeds with a very similar L:CHO ratio (1:2) have been proven to provide the best balance of carbohydrate and lipid when rearing juvenile *J. edwardsii*^(71)^. Therefore, based on the results presented, we recommend that high protein and moderate inclusion of carbohydrates must be considered when formulating feeds for *H. gammarus* juveniles. While relative growth benefits of lobsters fed the 500MED experimental diet did not exceed that of lobsters fed the control diet, it is worth considering that replacing fresh diets with extruded feeds can be advantageous in a cost-effective point of view. However, from an economic point of view, the ingredients used in the formulation of experimental extruded feeds were not the most sustainable. Future studies, following some of the recommendations presented here and using sustainable alternative ingredients (e.g. insect meal, industrial by-products), will be of extreme importance for the establishment of European lobster farming on a commercial scale.

## References

[1] Triantafyllidis A, Apostolidis AP, Katsares V, (2005) Mitochondrial DNA variation in the European lobster (*Homarus gammarus*) throughout the range. Mar Biol 146, 223–235.

[2] Agnalt AL, Van der Meeren GI, Jørstad KE, (1999) Stock enhancement of European lobster (*Homarus gammarus*): a large-scale experiment off south-western Norway (Kvitsøy). In Stock Enhancement and Sea Ranching, pp. 401–419 [B Howell, E Moksness & T Svåsand Eds.]. Farnham: Fishing New Book Ltd.

[3] Latrouite D & Lorec J (1991) L'experience Francaise de forcage du recruitement du homard Europeen (*Homarus gammarus*) resultants preliminaries. In *ICES Marine Science Symposia*, pp. 93–98.

[4] Cook W (1995) *A lobster stock enhancement experiment in Cardigan Bay*. Final report.

[5] Browne R & Mercer JP (1998) The European clawed lobster (*Homarus gammarus*) stock enhancement in the Republic of Ireland. In *Workshop on Lobster Stock Enhancement*, pp. 33–41. Quebec: Canadian Industry Report of Fisheries and Aquatic Sciences.

[6] Agnalt AL (2004) Enhancing the European lobster (*Homarus gammarus*) stock at Kvitsøy Islands: perspectives on rebuilding Norwegian stocks. In Stock Enhancement and Sea Ranching: Developments, Pitfals, and Opportunitties, pp. 415–426 [KM Leber, S Kitada, HL Blankenship, Eds.]. Oxford: Blackwell Publishing Ltd.

[7] Schmalenbach I, Mehrtens F, Janke M, (2011) A mark-recapture study of hatchery-reared juvenile European lobsters, *Homarus gammarus*, released at the rocky island of Helgoland (German Bight, North Sea) from 2000 to 2009. Fish Res 108, 22–30.

[8] Powell A (2016) New developments in European lobster aquaculture. Aquac Europe 41, 5–12.

[9] Daniels CL, Wills B, Ruiz-Perez M, (2015) Development of sea based container culture for rearing European lobster (*Homarus gammarus*) around South West England. Aquaculture 448, 186–195.

[10] Daniels CL, Merrifield DL, Ringø E, (2013) Probiotic, prebiotic and synbiotic applications for the improvement of larval European lobster (*Homarus gammarus*) culture. Aquaculture 416–417, 396–406.

[11] Drengstig A & Bergheim A (2013) Commercial land-based farming of European lobster (*Homarus gammarus* L.) in recirculating aquaculture system (RAS) using a single cage approach. Aquacult Eng 53, 14–18.

[12] Halswell P, Daniels CL & Johanning L (2018) Framework for evaluating external and internal parameters associated with Sea Based Container Culture (SBCC): towards understanding rearing success in European lobsters (*Homarus gammarus*). Aquacult Eng 83, 109–119. 10.1016/j.aquaeng.2018.09.005 PMC6472679 31007313

[13] Middlemiss KL, Daniels CL, Urbina MA, (2015) Combined effects of UV irradiation, ozonation, and the probiotic *Bacillus* spp. on growth, survival, and general fitness in European lobster (*Homarus gammarus*). Aquaculture 444, 99–107.

[14] Powell A, Hinchcliffe J, Sundell K, (2017) Comparative survival and growth performance of European lobster larvae, *Homarus gammarus*, reared on dry feed and conspecifics. Aquacult Res 48, 5300–5310.

[15] Gamble S, Pirozzi I, Hall MR, (2015) The effects of pre-digested protein sources on the performance of early-mid stage *Panulirus ornatus* phyllosoma. Aquaculture 440, 17–24.

[16] Floreto EAT, Bayer RC & Brown PB (2000) The effects of soybean-based diets, with and without amino acid supplementation, on growth and biochemical composition of juvenile American lobster, *Homarus americanus*. Aquaculture 189, 211–235.

[17] Crear BJ & Forteath GNR (2002) Feeding has the largest effect on the ammonia excretion rate of the southern rock lobster, *Jasus edwardsii*, and the western rock lobster, *Panulirus cygnus*. Aquacult Eng 26, 239–250.

[18] Goncalves R, Lund I, Gesto M, (2020) The effect of dietary protein, lipid, and carbohydrate levels on the performance, metabolic rate and nitrogen retention in juvenile European lobster (*Homarus gammarus*, L.). Aquaculture 525, 735334.

[19] Burton CA (2003) Lobster hatcheries and stocking programmes: an introductory manual. Seafish Report No. SR552.

[20] Wickins JF & Lee DO (2002) Crustacean Farming, Ranching and Culture. Oxford: Blackwell.

[21] Williams KC (2007) Feeds development for post-larval spiny lobster: a review. Bull Fish Res Agen 20, 25–37.

[22] Perera E & Simon C (2015) Digestive physiology of spiny lobsters: implications for formulated diet development. Rev Aquacult 7, 243–261.

[23] Williams KC (2007) Nutritional requirements and feeds development for post-larval spiny lobster: a review. Aquaculture 263, 1–14.

[24] Castell JD & Budson SD (1974) Lobster nutrition: the effect on *Homarus americanus* of dietary protein levels. J Fish Res Board Can 31, 1363–1370.

[25] Conklin DE, Devers K & Bordner C (1977) Development of artificial diets for the lobster, *Homarus americanus*. J World Aquac Soc 8, 841–852.

[26] Lim BK, Sakurai N, Sugihara T, (1997) Survival and growth of the American lobster *Homarus americanus* fed formulated feeds. Bull Mar Sci 61, 159–163.

[27] Tlusty MF, Fiore DR & Goldstein JS (2005) Use of formulated diets as replacements for Artemia in the rearing of juvenile American lobsters (*Homarus americanus*). Aquaculture 250, 781–795.

[28] Hinchcliffe J, Powell A, Langeland M, (2020) Comparative survival and growth performance of European lobster *Homarus gammarus* post-larva reared on novel feeds. Aquacult Res 51, 102–113.

[29] Tacon AGJ (1996) Nutritional studies in crustaceans and the problems of applying research findings to practical farming systems. Aquacult Nutr 2, 165–174.

[30] Albalat A, Johnson L, Coates CJ, (2019) The effect of temperature on the physiological condition and immune-capacity of European lobsters (*Homarus gammarus*) during long-term starvation. Front Mar Sci 6, 1–14.

[31] Yun H, Shahkar E, Katya K, (2016) Effects of bioflocs on dietary protein requirement in juvenile whiteleg shrimp, *Litopenaeus vannamei*. Aquacult Res 47, 3203–3214.

[32] Sacristán HJ, Di Salvatore P, Fernández-Gimenez AV, (2019) Effects of starvation and stocking density on the physiology of the male of the southern king crab *Lithodes santolla*. Fish Res 218, 83–93.

[33] Parslow-Williams PJ, Atkinson RJA & Taylor AC (2001) Nucleic acids as indicators of nutritional condition in the Norway lobster *Nephrops norvegicus*. Mar Ecol Prog Ser 211, 235–243.

[34] Schoo KL, Aberle N, Malzahn AM, (2014) The reaction of European lobster larvae (*Homarus gammarus*) to different quality food: effects of ontogenetic shifts and pre-feeding history. Oecologia 174, 581–594. 24072442 10.1007/s00442-013-2786-5

[35] Chícharo MA & Chícharo L (2008) RNA:DNA ratio and other nucleic acid derived indices in marine ecology. Int J Mol Sci 9, 1453–1471. 19325815 10.3390/ijms9081453 PMC2635731

[36] Labh SN, Prasad A & Ranjan R (2014) Effects of dietary protein concentrations on growth and RNA:DNA ratio of rainbow trout (*Oncorhynchus mykiss*) cultured in Nuwakot district of Nepal. Int J Fish Aquat Stud 1, 184–188.

[37] Glass HJ & Stark JR (1995) Carbohydrate digestion in the European lobster *Homarus gammarus* (L.). J Crust Biol 15, 424.

[38] Glass HJ & Stark JR (1994) Protein digestion in the European lobster, *Homarus gammarus* (L.). Comp Biochem Physiol B: Comp Biochem 108, 225–235.

[39] Simon CJ (2009) Digestive enzyme response to natural and formulated diets in cultured juvenile spiny lobster, *Jasus edwardsii*. Aquaculture 294, 271–281.

[40] Rötzer MAIN & Haug JT (2015) Larval development of the European lobster and how small heterochronic shifts lead to a more pronounced metamorphosis. Int J Zool 2015, 1–17.

[41] ISO 5983-2 (2005) Animal Feeding Stuffs – Determination of Nitrogen Content and Calculation of Crude Protein Content – Part 2: Block Digestion/Steam Distillation Method.

[42] Bligh EG & Dyer WJ (1959) A rapid method of total lipid extraction and purification. Can J Biochem Physiol 37, 911–917. 13671378 10.1139/o59-099

[43] NMKL 23 (1991) Gravimetric Determination in Meat and Meat Products.

[44] Rutherfurd SM (2009) Accurate determination of the amino acid content of selected feedstuffs. Int J Food Sci Nutr 60, 53–62. 18946798 10.1080/09637480802269957

[45] Larsen BK, Dalsgaard J & Pedersen PB (2012) Effects of plant proteins on postprandial, free plasma amino acid concentrations in rainbow trout (*Oncorhynchus mykiss*). Aquaculture 326–329, 90–98.

[46] Cuzon G & Guillaume J (1997) Energy and protein: energy ratio. In Crustacean Nutrition, pp. 51–70 [LR D'Abramo, DE Conklin, and DM Akiyama, editors]. Baton Rouge, LA: World Aquaculture Society.

[47] Nguyen NTB, Chim L, Lemaire P, (2014) Feed intake, molt frequency, tissue growth, feed efficiency and energy budget during a molt cycle of mud crab juveniles, *Scylla serrata* (Forskål, 1775), fed on different practical diets with graded levels of soy protein concentrate as main source of prote. Aquaculture 434, 499–509.

[48] Christie WW & Han X (2010) Lipid Analysis: Isolation, Separation, Identification and Lipidomic Analysis, 4th ed. Cambridge, UK: Elsevier Ltd.

[49] Caldarone EM, Wagner M, St Onge-Burns J, (2001) Protocol and guide for estimating nucleic acids in larval fish using a fluorescence microplate reader. Northeast Fisheries Science Center Reference Document 01-11, 22 p.

[50] Caldarone EM, Clemmesen CM, Berdalet E, (2006) Intercalibration of four spectrofluorometric protocols for measuring RNA/DNA ratios in larval and juvenile fish. Limnol Oceanogr Methods 4, 153–163.

[51] Rotllant G, Moyano FJ, Andrés M, (2008) Evaluation of fluorogenic substrates in the assessment of digestive enzymes in a decapod crustacean *Maja brachydactyla* larvae. Aquaculture 282, 90–96.

[52] Core Team R (2018) R: A Language and Environment for Statistical Computing. Vienna, Austria: R Foundation for Statistical Computing.

[53] Kasambara A & Mundt F (2020) factoextra: Extract and Visualize the Results of Multivariate Data Analyses.

[54] Grimm C, Lehmann K, Clemmesen C, (2015) RNA/DNA ratio is an early responding, accurate performance parameter in growth experiments of noble crayfish *Astacus astacus* (L.). Aquacult Res 46, 1937–1945.

[55] Simon CJ, Fitzgibbon QP, Battison A, (2015) Bioenergetics of nutrient reserves and metabolism in spiny lobster juveniles *Sagmariasus verreauxi*: predicting nutritional condition from hemolymph biochemistry. Physiol Biochem Zool 88, 266–283. 25860826 10.1086/681000

[56] Mente E, Houlihan DF & Smith K (2001) Growth, feeding frequency, protein turnover, and amino acid metabolism in European lobster *Homarus gammarus* L. J Exp Zool 289, 419–432. 11351329 10.1002/jez.1023

[57] Nelson MM, Bruce MP, Nichols PD, (2006) Nutrition of wild and cultured lobsters. In Lobsters: Biology, Management, Aquaculture and Fisheries, pp. 205–230 [BF Phillips Ed.]. Oxford: Blackwell Publishing Ltd.

[58] Cuzon G, Guillaume J & Cahu C (1994) Composition, preparation and utilization of feeds for Crustacea. Aquaculture 124, 253–267.

[59] Mente E (2006) Protein nutrition in crustaceans. CAB Rev 1, 1–7.

[60] Buckley L, Caldarone E & Ong TL (1999) RNA-DNA ratio and other nucleic acid-based indicators for growth and condition of marine fishes. Hydrobiologia 401, 265–277.

[61] Pilar Olivar M, Diaz MV & Alexandra Chícharo M (2009) Efecto del tipo de tejido sobre los cocientes ARN: ADN en larvas de peces marinos. Sci Mar 73, 171–182.

[62] Catalán IA, Berdalet E, Olivar MP, (2007) Response of muscle-based biochemical condition indices to short-term variations in food availability in post-flexion reared sea bass *Dicentrarchus labrax* (L.) larvae. J Fish Biol 70, 391–405.

[63] Lemos D, Garcia-Carreño F, Hernández P, (2002) Ontogenetic variation in digestive proteinase activity, RNA and DNA content of larval and postlarval white shrimp *Litopenaeus schmitti*. Aquaculture 214, 363–380.

[64] Moss SM (1994) Growth rates, nucleic acid concentrations, and RNA DNA ratios of juvenile white shrimp, *Penaeus vannamei* boone, fed different algal diets. J Exp Mar Biol Ecol 182, 193–204.

[65] Mattson J & Mykles DL (1993) Differential degradation of myofibrillar proteins by four calcium-dependent proteinase activities from lobster muscle. J. Exp. Zool. 265, 97–106.

[66] O'brien JJ & Skinner DM (1987) Characterization of enzymes that degrade crab exoskeleton: I. Two alkaline cysteine proteinase activities. J Exp Zool 243, 389–400.

[67] Lovett DL & Felder DL (1990) Ontogenetic change in digestive enzyme activity of larval and postlarval white shrimp *Penaeus setiferus* (Crustacea, Decapoda, Penaeidae). Biol Bull 178, 144–159. 29314933 10.2307/1541973

[68] Simon CJ & Jeffs AG (2013) The effect of dietary carbohydrate on the appetite revival and glucose metabolism of juveniles of the spiny lobster, *Jasus edwardsii*. Aquaculture 384–387, 111–118.

[69] Casillas Hernández R, Magallón F, Portillo G, (2002) La actividad de proteasa, amilasa y lipasa durante los estadios de muda del camarón azul *Litopenaeus stylirostris*. Rev Investig Mar 23, 35–40.

[70] Barker PL & Gibson R (1977) Observations on the feeding mechanism, structure of the gut, and digestive physiology of the European lobster *Homarus gammarus* (L.) (Decapoda: Nephropidae). J Exp Mar Biol Ecol 26, 297–324.

[71] Johnston DJ, Calvert KA, Crear BJ, (2003) Dietary carbohydrate/lipid ratios and nutritional condition in juvenile southern rock lobster, *Jasus edwardsii*. Aquaculture 220, 667–682.

